# Multidrug-resistant and extended-spectrum beta-lactamase-producing uropathogens in children in Bhaktapur, Nepal

**DOI:** 10.1186/s41182-020-00251-6

**Published:** 2020-08-03

**Authors:** Ganendra Bhakta Raya, Bhim Gopal Dhoubhadel, Dhruba Shrestha, Sunayana Raya, Ujjwal Laghu, Ashok Shah, Bijendra Bhakta Raya, Rita Kafle, Christopher M. Parry, Koya Ariyoshi

**Affiliations:** 1 Siddhi Memorial Hospital, Bhaktapur, Nepal; 2 Department of Clinical Medicine, Institute of Tropical Medicine, Nagasaki University, 1-12-4 Sakamoto, Nagasaki, 852-8523 Japan; 3 School of Tropical Medicine and Global Health (TMGH), Nagasaki University, 1-12-4 Sakamoto, Nagasaki, 852-8523 Japan; 4 German Nepal Tuberculosis Project (GENETOP), Kathmandu, Nepal; 5 Kathmandu Medical College, Kathmandu, Nepal; 6 Liverpool School of Tropical Medicine, Liverpool, UK

**Keywords:** Urinary tract infection, Multidrug-resistance, Extended-spectrum beta-lactamase, *E. coli*, *K. pneumoniae*, Children, Nepal

## Abstract

**Background:**

The emergence of multidrug-resistant (MDR) and extended-spectrum beta-lactamase (ESBL)-producing uropathogens has complicated the treatment of urinary tract infections (UTI). Paediatric UTI is a common illness, which if not treated properly, may lead to acute and long-term complications, such as renal abscess, septicaemia, and renal scarring. This study aimed to determine the prevalence of MDR and ESBL-producing uropathogens among children.

**Methods:**

During the study period (April 2017–April 2018), midstream urine samples were collected following aseptic procedures from children < 16 years in Siddhi Memorial Hospital. Standard culture and biochemical tests were performed to identify uropathogens and antimicrobial susceptibility test was done by modified Kirby-Bauer disc diffusion method following Clinical and Laboratory Standard Institute (CLSI) guidelines. ESBL-producing uropathogens were screened by ceftazidime (30 μg) and cefotaxime (30 μg) discs, and confirmed by the combination disc tests: ceftazidime + clavulanic acid (30/10 μg) or cefotaxime + clavulanic acid (30/10 μg) as recommended by CLSI.

**Results:**

We processed 5545 non-repeated urine samples from the children with symptoms of UTI. A significant growth of uropathogens was observed in 203 samples (3.7%). The median age of the children was 24 months (interquartile range (IQR), 12–53 months). *Escherichia coli* (*n* = 158, 77.8%) and *Klebsiella pneumoniae* (*n* = 30, 14.8%) were common among the uropathogens. Among them, 80.3% were resistant to amoxycillin and 51.2% were resistant to cotrimoxazole. Most of them were susceptible to amikacin, nitrofurantoin, and ofloxacin. MDR was detected in 34.5% (*n* = 70/203) and ESBL producers in 24.6% (*n* = 50/203) of them. The proportion of MDR isolates was higher in children < 5 years (*n* = 59/153, 38.6%) than children ≥ 5 years (*n* = 11/50, 22%) (*P* = 0.03).

**Conclusions:**

Nitrofurantoin, ofloxacin, and amikacin can be used for the empirical treatment for UTI in children in Bhaktapur, Nepal. MDR and ESBL-producing uropathogens are prevalent; this warrants a continuous surveillance of antimicrobial resistance.

## Introduction

Urinary tract infection (UTI) is a common cause of illness in children. Gastrointestinal bacteria, such as *Escherichia coli* and *Klebsiella pneumoniae*, cause UTIs. Effective antimicrobial treatment of these infections is necessary; if untreated, they may complicate to renal abscess, septicaemia, renal scarring, and renal failures which can be life threatening in children [1, 2]. Not all UTI cases can be diagnosed with an isolation of a uropathogen and its antimicrobial susceptibility can be known; therefore, understanding the distribution of uropathogens and their resistance patterns will help the doctors and caregivers choose appropriate antibiotics for an empirical treatment [3].

The growing prevalence of antimicrobial resistance among uropathogens is an enormous concern. The increase in proportion of multidrug-resistance (MDR) and ESBL-producing uropathogens in children has not only limited the treatment by rendering the first-line antibiotics ineffective but also complicated the treatment by increasing the hospital stay. The problem of antimicrobial resistance has become worse in resource-limited countries where antimicrobials are available over-the-counter [4–6]. Surveillance of resistance patterns in these countries is thus vital to help doctors and caregivers prescribe effective treatment to patients. Such surveillance also informs policy makers to develop public health measures that may help decrease the prevalence of resistant organisms, which may decrease economic burden in the community [7–9].

In this study, we aimed to determine the prevalent uropathogens, their antimicrobial susceptibility, and the prevalence of MDR and ESBL producers in children attending a paediatric hospital in Nepal with symptoms of UTI.

## Methods

### Study design and setting

We carried out this cross-sectional study from April 14, 2017 to April 13, 2018, in the Department of Microbiology, Siddhi Memorial Hospital (SMH) in Bhaktapur, Nepal. SMH is a 50-bedded hospital that provides tertiary care to children. We included children less than 16 years of age in this study.

### Laboratory methods

We collected urine specimens following aseptic procedures, such as midstream urine, catheterisation, and suprapubic aspiration from children with clinical symptoms suggestive of a possible UTI. We transported the urine samples to the laboratory immediately and processed by the semi-quantitative streaking method. We inoculated 10 μL of urine onto a cysteine lactose electrolyte deficient (CLED) agar plate using a sterile calibrated wired loop. Inoculated plates were incubated at 37 °C in aerobic conditions for 18 h. We diagnosed a UTI when we observed a significant growth (≥ 10^5^colony-forming units/mL) of a single organism. To isolate and identify the organisms, we performed standard microbiological methods including colony morphology, Gram staining, catalase, oxidase, and in-house set of biochemical tests [10].

### Antimicrobial susceptibility testing

We used the modified Kirby-Bauer method to determine the susceptibility of bacterial isolates on Mueller Hinton agar (Oxoid, UK) following the guidelines of the Clinical and Laboratory Standards Institute (CLSI), Wayne, USA [11]. We tested common antimicrobials that we prescribe in our hospital by using these antimicrobial discs: amoxycillin (10 μg), amikacin (30 μg), cefazolin (30 μg), cefotaxime (30 μg), ceftazidime (30 μg), cotrimoxazole (1.25/23.7), ofloxacin (5 μg), and nitrofurantoin (300 μg). We assigned the results of susceptibility tests susceptible or resistant according to the zone size standards of CLSI. We defined MDR isolate if it showed resistance to at least one antimicrobial from the three groups of the first-line antimicrobials: beta-lactam, aminoglycoside, quinolone, sulphonamide, and nitrofuran.

### Screening and confirmation of ESBL-producing isolates

We screened all Gram-negative bacilli for ESBL production. We tested the susceptibilities to third-generation cephalosporins by using ceftazidime (30 μg) and cefotaxime (30 μg) discs. As recommended by CLSI, we considered the isolates as potential ESBL producers if the zone of inhibition was ≤ 22 mm for ceftazidime or ≤ 27 mm for cefotaxime. The potential ESBL producers were then examined for confirmation by the combination disc test as the CLSI guideline. In this test, we tested the isolates against a ceftazidime (30 μg) disc and a ceftazidime + clavulanic acid (30/10 μg) combination disc, and we compared the inhibition zones around these discs. We considered that the test was positive for the confirmation of ESBL producers if the inhibition zone diameter was ≥ 5 mm larger with the combination disc than the ceftazidime disc. Similarly, we also performed the confirmation test with a cefotaxime (30 μg) disc and a cefotaxime + clavulanic acid (30/10 μg) combination disc in all potential isolates; the results were interpreted as positive if the inhibition zone diameter was ≥ 5 mm larger with the combination disc than the cefotaxime disc. Having positive results in any one of these two confirmatory tests, we confirmed the isolates as ESBL producers.

### Data analysis

We analysed the data in Stata 14 (Stata Corp, Texas). The chi-squared test was used to compare the proportions of isolates between males and females, and between younger than 5 years and equal or older than 5 years.

## Results

**Fig. 1:**
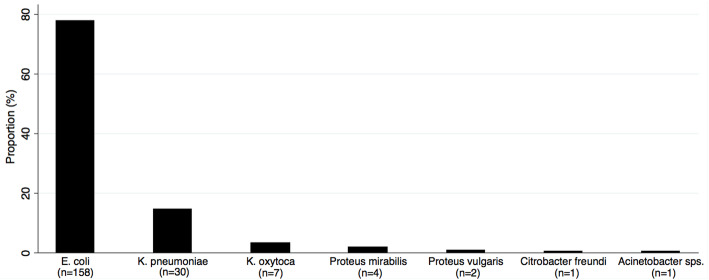
Distribution of uropathogens in children in Siddhi Memorial Hospital, Bhaktapur, Nepal. The bar graph shows the proportion of the seven types of uropathogens isolated (*n* = 203) from 5545 non-repeated urine samples. *E. coli* (77.8%) and *K. pneumoniae* (14.8%) were the predominant uropathogens isolated

The total number of urine samples processed during the study period was 5545. The number of isolates with the significant growth was 203. The prevalence of UTIs was 3.7% among these children. The median age of the children was 24 months (IQR, 12–53 months) and there were 101 male and 102 female children. Three-quarters (*n* = 153, 75.3%) of isolates were from the children less than 5 years. *E. coli* was the most common isolated uropathogen (*n* = 158, 77.8%) followed by *K. pneumoniae* (*n* = 30, 14.8%) (Fig. 1). The proportion of *E. coli* in female children was 83.3% and 72.2% (*P* = 0.05) in male children. There were no significant differences in the proportions of bacterial isolates between males and females, and between children less than 5 years and the older-aged groups (Table 1).

**Table 1:** Distributions of uropathogens by age and sex of children in Siddhi Memorial Hospital, Bhaktapur, Nepal

Uropathogens	Sex		Age	
	Male, no. (%) (*n* = 101)	Female, no. (%) (*n* = 102)	< 5 years, no. (%) (*n* = 153)	≥ 5 years, no. (%) (*n* = 50)
*E. coli*	73 (72.2)	85 (83.3)	118 (77.1)	40 (80.0)
*K. pneumoniae*	19 (18.8)	11 (10.8)	24 (15.7)	6 (12.0)
*K. oxytoca*	4 (4.0)	3 (2.9)	5 (3.2)	2 (4.0)
*P. mirabilis*	4 (4.0)	0 (0.0)	4 (2.6)	0 (0.0)
*P. vulgaris*	1 (1.0)	1 (1.0)	1 (0.7)	1 (2.0)
*C. freundii*	0 (0.0)	1 (1.0)	0 (0.0)	1 (2.0)
*Acinetobacter* spp*.*	0 (0.0)	1 (1.0)	1 (0.7)	0 (0.0)

**Fig. 2:**
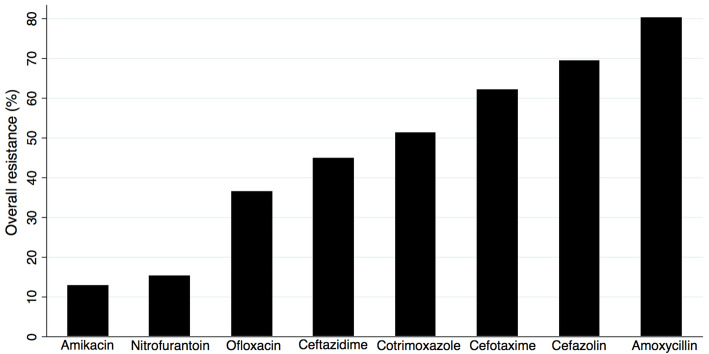
Antimicrobial resistance patterns of uropathogens in children in Siddhi Memorial Hospital, Bhaktapur, Nepal. The bar graph shows the proportion of uropathogens that showed antimicrobial resistance to commonly used eight antimicrobials in the hospital. The uropathogens were most susceptible to amikacin and nitrofurantoin and resistant to cefotaxime, cefazolin, and amoxycillin

We show the antimicrobial resistance patterns of the uropathogens in Table 2. *E. coli* was most susceptible to nitrofurantoin and amikacin, whereas *K. pneumoniae* was most susceptible to amikacin and ofloxacin. A high proportion of the uropathogens was resistant to amoxycillin, cefazolin, cefotaxime, and cotrimoxazole, whereas amikacin, nitrofurantoin, and ofloxacin were the antimicrobials to which most uropathogens were susceptible (Fig. 2).

**Table 2:** Antimicrobial resistance patterns of uropathogens in children in Siddhi Memorial Hospital, Bhaktapur, Nepal

Antimicrobials	Uropathogens						
	*E. coli*, no. (%) (*n* = 158)	*K. pneumoniae*, no. (%) (*n* = 30)	*K. oxytoca*, no. (%) (*n* = 7)	*P. mirabilis*,no. (%) (*n* = 4)	*P. vulgaris*, no. (%) (*n* = 2)	*C. freundii*, no. (%) (*n* = 1)	*Acinetobacter* spp., no. (%) (*n* = 1)
Amikacin	22 (13.9)	2 (6.7)	1 (14.3)	1 (25)	0 (0)	0 (0)	0 (0)
Amoxycillin	126 (79.7)	28 (93.3)	5 (71.4)	2 (50)	1 (50)	1 (100)	0 (0)
Cefazolin	111 (70.3)	20 (66.7)	5 (71.4)	2 (50)	1 (50)	1 (100)	1 (100)
Cefotaxime	103 (65.2)	19 (63.3)	2 (28.6)	0 (0)	1 (50)	0 (0)	1 (100)
Ceftazidime	75 (47.5)	15 (50)	1 (14.3)	0 (0)	0 (0)	0 (0)	0 (0)
Cotrimoxazole	82 (51.9)	18 (60)	2 (28.6)	1 (25)	1 (50)	0 (0)	0 (0)
Ofloxacin	66 (41.8)	7 (23.3)	0 (0)	1 (25)	0 (0)	0 (0)	0 (0)
Nitrofurantoin	9 (5.7)	13 (43.3)	2 (28.6)	4 (100)	2 (100)	0 (0)	1 (100)

**Fig. 3:**
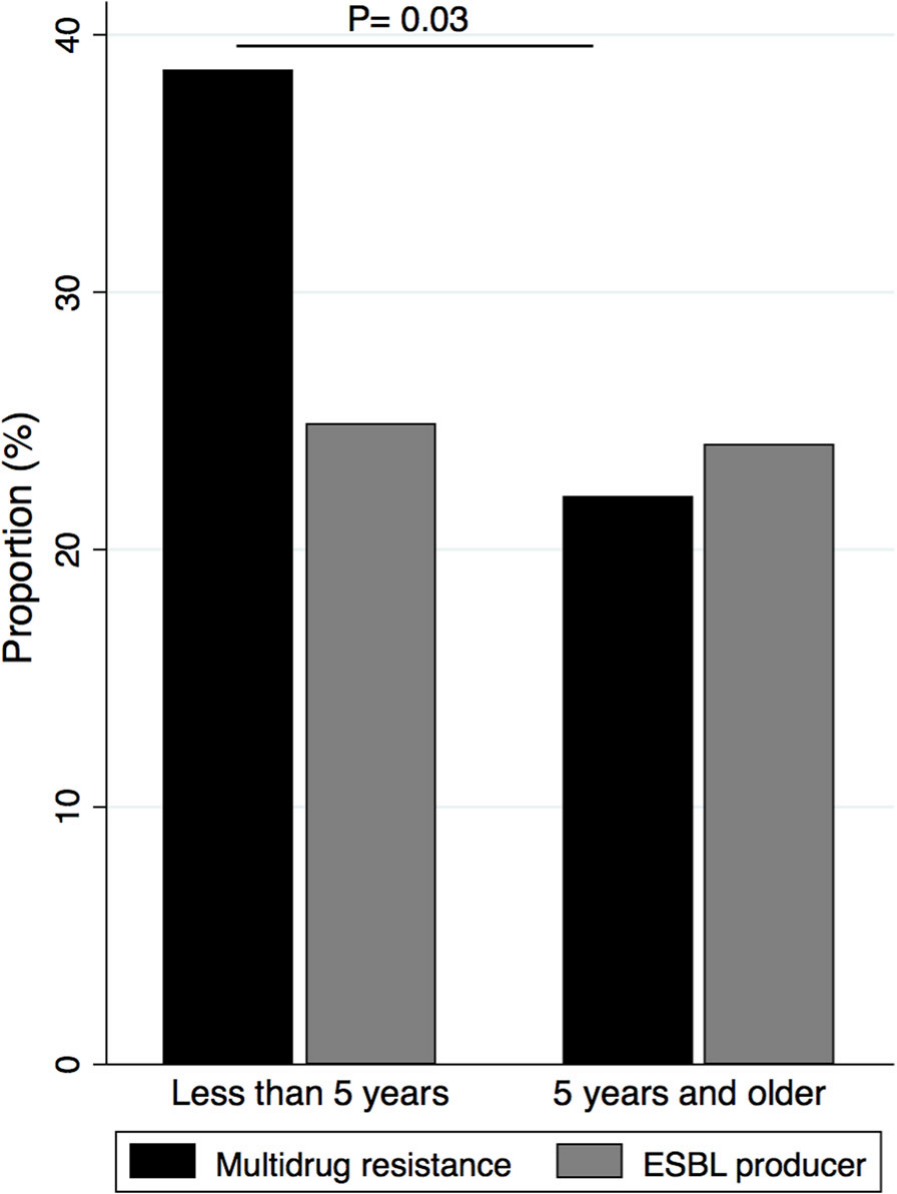
Distributions of MDR and ESBL-producing uropathogens in children. The bar graph shows the comparison of the proportions of MDR and ESBL-producing uropathogens between children < 5 years old and ≥ 5 years old. The proportions of MDR were 38.6% (*n* = 59/153) in < 5 years and 22% (*n* = 11/50) in ≥ 5 years old children (*P* = 0.03)

We detected multidrug-resistance in 34.5% (*n* = 70/203) of the uropathogens. The proportions of MDR in male and female children were similar (*n* = 34/101, 33.7% in male vs *n* = 36/102, 35.3% in female children) (*P* = 0.8). We observed a higher proportion of MDR in children younger than 5 years (*n* = 59/153, 38.6%) than older aged group (*n* = 11/50, 22%) (*P* = 0.03) (Fig. 3). We detected ESBL producers in 24.6% (*n* = 50/203) of the uropathogens. The proportions of ESBL producers were not different between male (*n* = 28/101, 27.7%) and female children (*n* = 22/102, 21.6%) (*P* = 0.3), and between children under 5 years (*n* = 38/153, 24.8%) and older-aged children (*n* = 12/50, 24%) (*P* = 0.9). MDR was detected in 34.2% of *E. coli* and 36.7% of *K. pneumoniae* isolates. ESBL producers were detected in 27.2% of *E. coli* and 23.3% of *K pneumoniae* isolates (Table 3).

**Table 3:** Prevalence of MDR and ESBL-producing uropathogens in children in Siddhi Memorial Hospital, Bhaktapur, Nepal

Uropathogens	MDR (%)	ESBL (%)
*E. coli* (*n* = 158)	54 (34.2)	43 (27.2)
*K. pneumoniae* (*n* = 30)	11 (36.7)	7 (23.3)
*K. oxytoca* (*n* = 7)	2 (28.6)	0 (0)
*P. mirabilis* (*n* = 4)	2 (50)	0 (0)
*P. vulgaris* (*n* = 2)	1 (50)	0 (0)
*C. freundii* (*n* = 1)	0 (0)	0 (0)
*Acinetobacter* spp*.* (*n* = 1)	0 (0)	0 (0)
Total (*n* = 203)	70 (34.5)	50 (24.6)

The data are presented as a number (proportion, %) of uropathogens that were MDR and ESBL producer

## Discussion

This study shows that *E. coli* and *K. pneumoniae* were common uropathogens in the children who attended to Siddhi Memorial Hospital, Bhaktapur, Nepal. Most of the uropathogens were resistant to the commonly prescribed antimicrobials, such as amoxycillin and cotrimoxazole, that render these antimicrobials ineffective for the empirical treatment. Multidrug-resistance and ESBL producers were detected in high proportions, and MDR was more common in children younger than 5 years than older children.

Urinary tract infections are caused by Gram-positive and Gram-negative bacteria, and fungi [9]. *E. coli* and *K. pneumoniae* are the common causes of UTI in children and adults [9, 12]. In this study, all the isolated uropathogens were Gram-negative bacteria, 77.8% were *E. coli* and 14.8% were *K. pneumoniae*, together they comprised 92.6% of all the uropathogens. These findings were comparable with the studies from Turkey, Ethiopia, Iran, and Nepal [12–15].

Uropathogens generally reside in the gut. When the periurethral region is contaminated with these bacteria, a UTI starts by the development of colonisation of urethra and migration of the bacteria to the bladder [9]. In lack or failure of treatment, a UTI can result in serious sequelae that include renal abscess, renal scarring, and chronic renal failure [1, 2].

The prevalence of antimicrobial resistance to first-line antimicrobials for UTIs in children has increased in resource-limited countries. A systematic review on 58 observation studies shows the pooled prevalence of resistance in *E. coli* is higher—79.8% to ampicillin, 60.3% to co-amoxiclav, and 26.8% to ciprofloxacin—in non-OECD (Organisation for Economic Co-operation and Development) countries than the resistance of *E. coli—*53.4% to ampicillin, 8.2% to co-amoxiclav, and 2.1% to ciprofloxacin—in OECD countries [4]. In this study, we found that 80.3% of isolates were resistant to amoxycillin, 62.1% resistant to cefotaxime, and 51.2% resistant to cotrimoxazole. These are the first-line treatment of UTIs in children in Nepal. One of the reasons for the high prevalence of resistance to routine antibiotics is the availability of over-the-counter antimicrobials [4]. Because of the high prevalence of resistant uropathogens, the first-line antimicrobials, such as amoxycillin and cotrimoxazole, may be ineffective and only a few antimicrobials are left for effective treatment of UTIs. Our study showed that most uropathogens, which are community-acquired, in Bhaktapur, Nepal, were susceptible to nitrofurantoin, ofloxacin, and amikacin. Amoxycillin and cotrimoxazole, which are inexpensive, easily available, and have fewer side effects, can no longer be prescribed as an empirical treatment in this region due to the high level of antimicrobial resistance acquired by the uropathogens.

Increasing resistance of uropathogens to three or more groups of antimicrobials (MDR) has become a severe threat to the health care system [16]. Gram-negative bacteria that cause UTIs have developed resistance to many drug classes, including beta-lactams, aminoglycoside, and quinolones. In this study, we found 34.2% of *E. coli* and 36.7% of *K. pneumoniae* were MDR. Our findings were similar to those of Shrestha et al. who reported that 34% isolates were MDR in Dharan, a city in the eastern part of Nepal, in 2018 [17]. However, few studies in Kathmandu showed a higher proportion (52.3%, 64.9%) of MDR than our study in *E. coli* [12, 18]. The higher prevalence of MDR in Kathmandu than Bhaktapur may be due to bigger general hospitals in Kathmandu, where children and adults with more risk factors for MDR are treated, than in Bhaktapur. Previous antimicrobial use, previous hospitalisation, urinary catheterisation, and urinary tract anomalies are some of the known risk factors for MDR [19, 20]. We found that MDR was significantly more prevalent in children younger than 5 years than older children. One plausible explanation for this observation can be that the children younger than 5 years receive antimicrobials more often for repeated infections, such as acute respiratory infections and gastrointestinal infections, than the older children, which help the bacteria to develop resistance to multiple antimicrobials.

Infections with ESBL-producing organisms in children are associated with longer hospital stays, frequent complications, and increased mortality [21]. ESBL-producing Enterobacteriaceae causes one in seven (14%) UTI in children [6]. In our study, 24.6% of uropathogens were ESBL producers. Some other studies in Nepal also show a high prevalence of ESBL producers, 40% in Dharan and 38.9% in Kathmandu [17, 18]. This discrepancy of prevalence of ESBL producers may be due to the studies that were carried out in general hospitals where complicated UTI cases, including adult patients, were treated and are located in different geographical regions [6, 21]. Overall, these studies show a high prevalence of ESBL producers among uropathogens in Nepal.

This study has limitations. This study was conducted in one hospital for a period of 1 year, so the findings may not be generalisable. We could not evaluate the risk factors and outcomes of MDR and ESBL producers. The genotypes of ESBL producers could not be determined.

## Conclusions

We found a high prevalence of MDR and ESBL-producing uropathogens in children in Bhaktapur, Nepal. The prevalent uropathogens were susceptible to nitrofurantoin, ofloxacin, and amikacin, some of which may be used for the empirical treatment of UTIs in children. Improved antimicrobial stewardship in hospitals and a restricted use of over-the-counter antimicrobials maybe some of the ways to decrease the prevalence of antimicrobial resistance in the community.

## Data Availability

De-identified data of this study are available upon reasonable request from the corresponding author.

## Abbreviations

CLSI: Clinical and Laboratory Standards Institute
ESBL: Extended-spectrum beta-lactamase
MDR: Multidrug-resistant
SMH: Siddhi Memorial Hospital
UTI: Urinary tract infection
